# Predictors of Outcomes in Prurigo Nodularis in Patients Living With HIV: A Scoping Review

**DOI:** 10.1177/12034754241274345

**Published:** 2024-08-28

**Authors:** Shanti Mehta, Dea Metko, Kyle Seigel, Dalia Shamma, Vincent Piguet, David Croitoru

**Affiliations:** 1 Temerty Faculty of Medicine, University of Toronto, Toronto, ON, Canada; 2 Michael G. DeGroote School of Medicine, McMaster University, Hamilton, ON, Canada; 3 Division of Dermatology, Department of Medicine, University of Toronto, Toronto, ON, Canada; 4 Division of Dermatology, Department of Medicine, Women’s College Hospital, Toronto, ON, Canada; 5 Division of Dermatology, Department of Medicine, University Health Network, Toronto, ON, Canada

**Keywords:** HIV, immunodeficiency, virus, prurigo nodularis

To the Editor:

Patients living with HIV (PLHIV) are at risk for developing prurigo nodularis (PN), a chronic inflammatory and neuropathic dermatosis characterized by intensely itchy and hardened nodules.^1^ PN ranks among the most prevalent mucocutaneous diseases associated with HIV, affecting approximately 13.8% of PLHIV.^2^ While HIV and PN have been thoroughly explored independently, predictors of PN outcomes in PLHIV is poorly understood. Thus, our study aims to systematically review existing evidence, consisting of case reports, retrospective chart reviews, descriptive correlational, retrospective analysis, cross-sectional, and prospective studies, on predictors of outcomes in PN in PLHIV to identify consistently associated factors of risk and prognosis.

We searched MEDLINE and Embase using keywords related to HIV and PN per PRISMA guidelines^3^ (Supplemental Table S1). To be eligible for inclusion, results had to be peer-reviewed articles available in the English language. A total of 13 studies encompassing 522 patients were included in the review, comprising all study types mentioned above (Supplemental Figure S1). Study quality was assessed using the Joanna Briggs Institute critical appraisal tool (Supplemental Table S2).^4^

The average CD4+ T-cell reported was 88.2 cells/mm^3^ (n = 151/522), indicative of advanced immunosuppression. The average viral load reported was 82,583 copies/mL (n = 3/522). Treatment of HIV with highly active antiretroviral therapy (HAART) was reported in 342 (65.6%) patients. In one case report patients not receiving HAART with CD4-positive cell counts below 200 cells/mm^3^ were found to exhibit an increased prevalence of PN when compared to those receiving HAART (7.9% vs 3.4%; *P* = .01).^5^ In a case where 2 patients’ PN was refractory to HAART, the addition of raltegravir to regiment dramatically improved PN in both patients.^1^ In most cases, PN improvement was observed within 2 months of commencing HAART.^2^

Reported therapeutics used in PN treatment include topical therapies, specifically topical corticosteroids (n = 13, 2.49%), ultraviolet-B phototherapy (n = 12, 2.30%) and systemic therapies including corticosteroids, thalidomide, and antihistamines (n = 13, 2.49%). Comorbidities were reported in 326 patients (62.5%) and exhibited wide variability, ranging from psychiatric to cardiovascular and infectious diseases (Supplemental Tables S3 and S4). While the exact role of each comorbidity in PN occurrence and treatment in PLHIV remains unclear, it can be suggested that the presence of comorbidities may complicate treatment plans, potentially worsening the prognosis of PN in PLHIV. Positive HIV status poses an additional consideration prior to starting systemic immunosuppressants or biologics to treat PN.

We provided an overview of important predictors of PN outcomes in PLHIV. Together, the general trend of the literature suggests that PN improves concomitantly with HAART use for HIV, improvements in CD4 counts and viral load. This may be explained by a reduction in inflammation, improvement in immune function, and alterations in the cytokine landscape.^1,2^ Dermatologic treatments, specifically phototherapy, topicals and systemic therapies should be included in PN treatment strategies. The management of PN in PLHIV remains challenging due to the recalcitrant nature of the condition, regardless of HIV status. Limitations of this study include a small sample size and unclear risk of bias. Future research is crucial to understand the influence of comorbidities on PN prevalence in PLHIV to optimize management strategies.

## Supplemental Material

sj-docx-1-cms-10.1177_12034754241274345 – Supplemental material for Predictors of Outcomes in Prurigo Nodularis in Patients Living With HIV: A Scoping Review Supplemental material, sj-docx-1-cms-10.1177_12034754241274345 for Predictors of Outcomes in Prurigo Nodularis in Patients Living With HIV: A Scoping Review by Shanti Mehta, Dea Metko, Kyle Seigel, Dalia Shamma, Vincent Piguet and David Croitoru in Journal of Cutaneous Medicine and Surgery
