# Supplementary material for: Predictors of Outcomes in Prurigo Nodularis in Patients Living With HIV: A Scoping Review
Source: J Cutan Med Surg. 2024 Aug 28;28(6):601–2. doi: 10.1177/12034754241274345 (PMC11585177; doi:10.1177/12034754241274345)
Supplement: sj-docx-1-cms-10.1177_12034754241274345 – Supplemental material for Predictors of Outcomes in Prurigo Nodularis in Patients Living With HIV: A Scoping Review [file sj-docx-1-cms-10.1177_12034754241274345.docx]

**Supplemental Material**

**Table S1.** Search strategy.
Database:

**Embase** <1974 to 2024 January 05>

| **#** | **Query** | **Results from 8 Jan 2024** |
| --- | --- | --- |
| 1 | (prurigo nodularis or chronic pruritus or Nodular prurigo).mp. | 2,463 |
| 2 | exp human immunodeficiency virus/ or HIV.mp. | 506,375 |
| 3 | exp acquired immunodeficiency syndrome/ or AIDS.mp. | 684,248 |
| 4 | 2 or 3 | 1,022,616 |
| 5 | 1 and 4 | 120 |

**Ovid MEDLINE(R) and Epub Ahead of Print, In-Process, In-Data-Review & Other Non-Indexed Citations, Daily and Versions** <1946 to January 05, 2024>

| **#** | **Query** | **Results from 8 Jan 2024** |
| --- | --- | --- |
| 1 | (prurigo nodularis or chronic pruritus or Nodular prurigo).mp. | 1,434 |
| 2 | exp human immunodeficiency virus/ or HIV.mp. | 408,643 |
| 3 | exp acquired immunodeficiency syndrome/ or AIDS.mp. | 240,301 |
| 4 | 2 or 3 or 4 | 519,948 |
| 5 | 1 and 5 | 43 |

**Figure S1.** Flow diagram of literature screening using the Preferred Reporting Items for Systematic reviews and Meta-Analyses extension for Scoping Reviews (PRISMA-ScR


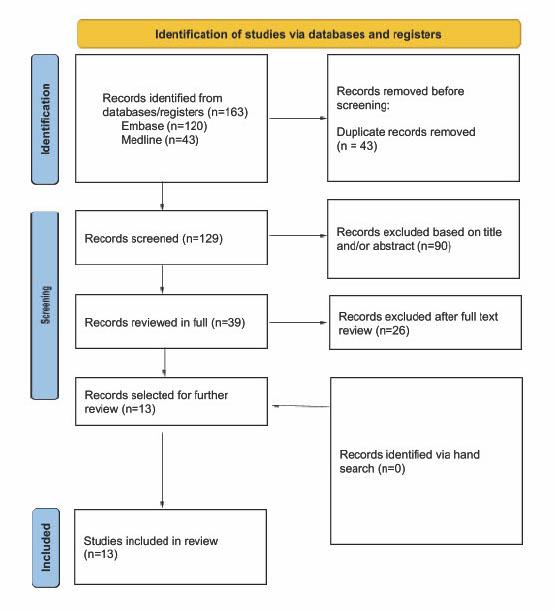


**Table S2:** Summary of included literature

| **Author, year of publication** | **Aim of study** | **Study design** | **Sample size** | **Results/summary** | **Risk of bias** |
| --- | --- | --- | --- | --- | --- |
| Bender 2019 | To examine associated dermatologic conditions in a large population of HIV patients at a tertiary care center with a diverse patient census | Retrospective chart review | 133 | Several dermatologic conditions were significantly more common in PLHIV than in the general population, including prurigo nodularis (p<0.0009, OR 11.44) | NA |
| Berger 1995 | To demonstrate the value of thalidomide in treating PN in AIDS, especially when photosensitivity is the basis of the PN | Case report | 1 | UVB therapy precipitated but multiple nodular excoriated papules and plaques with marked depigmentation developed on the trunk, scalp, and extremities | Moderate |
| Boozalis 2018 | To examine race and comorbidity trends in PN patients | Retrospective chart review | 133 | Black patients with PN were 10.5 times more likely to have HIV than race-matched controls with atopic dermatitis, and eight times more likely to have HIV than black patients with psoriasis | NA |
| Freytes 2007 | To determine the frequency of skin disease in persons infected HIV | Descriptive correlational | 8 | PN (P =0.0096) was associated with having a low CD4 cell count, and is one of the most frequent skin conditions in PLHIV | NA |
| Herranz 1998 | To report the case of an HIV positive patient who develops PN | Case report | 1 | Thalidomide successfully resolved PN in a patient with HIV | Moderate |
| Huang 2011 | To investigate the relationship between skin lesions and immune function in a HIV patient with and without HAART | Retrospective chart review | 94 | PN (13.8%) was one of the most common comorbidities in Chinese patients with HIV | NA |
| Jing 1999 | To investigate mucocutaneous manifestations of HIV infection | Retrospective analysis | 43 | The analysis showed that 71.7% of PLHIV had mucocutaneous disorders, with 29.7% having PN | NA |
| Kundu 1995 | To report the case of a man who presented with PN, and was concurrently diagnosed with HIV | Case report | 1 | Topical steroids were ineffective at treating PN in a patient with HIV, rather UVB phototherapy was more effective | Unclear |
| Magand 2011 | To compare the level of immunosuppression of patients diagnosed as HIV-positive after consulting for PN or herpes zoster in French Guiana | Retrospective chart review | 50 | The positive predictive value or HIV positivity was 36% for PN, with a positive predictive value of having a CD4 count <200/mm^3^ being 72% | NA |
| Maurer 2004 | To evaluate safety and efficacy of thalidomide in the treatment of PN in a group of HIV infected patients whose condition was recalcitrant to standard treatment | Prospective | 8 | Thalidomide reduced the signs and symptoms of PN in PLHIV | NA |
| Motegi 2014 | To report a case of Persistent PN in HIV-infected patient responsive to antiretroviral therapy with raltegravir | Case report | 1 | Within 2 months of starting ART, most of the multiple PN nodules had disappeared with post inflammatory pigmentation | Low |
| Unemori 2010 | To report cases of PN that dramatically improved within 2 weeks of initiating oral dosing with raltegravir | Case report | 2 | Raltegravir was highly effective in the recession of PN, possibly due to to reduction of viremia | Low |
| Queiroz Zancanaro 2005 | To evaluate the use of HAART on the prevalence and spectrum of cutaneous manifestations in HIV-infected patients | Cross sectional | 46 | Patients with CD4-positive cell counts less than 200 cells/mm^3^ had an increased prevalence PN, and patients not receiving HAART had increased rates PN | Low |

**Legend:** HIV, human immunodeficiency virus; PLHIV, people living with human immunodeficiency virus; PN, prurigo nodularis; AIDS, acquired immunodeficiency syndro

**Table S3**: Significant prognostic factors for patient outcomes

| **Prognostic Factor reported** | | | **Patients with reported variable *n (%)*** | **Unique studies with reported variable *n (%)*** |
| --- | --- | --- | --- | --- |
| **Total** | | | 522 (100) | 13 (100) |
| **Patient** | | |  |  |
| Age | | 522 (100) | 13 (100) |  |
| Sex | | 522 (100) | 13 (100) |  |
| Race | | 427 (81.8) | 12 (92.3) |  |
| **HIV** | | |  |  |
| CD4 count | | 254 (48.7) | 9 (69.2) |  |
| Viral load | | 64 (12.3) | 4 (30.8) |  |
| Treatment - HAART | | 342 (65.6) | 7 (53.8) |  |
| **Prurigo Nodularis** | | |  |  |
| Presentation | | 151 (28.9) | 7 (53.8) |  |
| Morphology | 54 (10.3) | 5 (38.5) |  |  |
| Location | 13 (2.49) | 5 (38.5) |  |  |
| Treatment – Systemic | | 13 (2.49) | 5 (38.5) |  |
| Thalidomide | 5 (0.958) | 4 (30.8) |  |  |
| Corticosteroids | 3 (0.575) | 2 (15.4) |  |  |
| Antihistamines | 11 (2.11) | 3 (23.1) |  |  |
| Treatment – Topical | | 13 (2.49) | 5 (38.5) |  |
| Corticosteroids | 13 (2.49) | 5 (38.5) |  |  |
| Treatment – Phototherapy | | 12 (2.30) | 4 (30.8) |  |
| **Other** | | |  |  |
| Comorbidities | | 326 (62.5) | 6 (46.2) |  |
| Neoplastic | NR | 2 (15.4) |  |  |
| Infectious | NR | 4 (30.8) |  |  |
| Inflammatory | NR | 2 (15.4) |  |  |
| Dermatological | NR | 2 (15.4) |  |  |
| Psychiatric | NR | 2 (15.4) |  |  |
| Cardiovascular | NR | 4 (30.8) |  |  |
| Other | NR | 2 (15.4) |  |  |
|  | | |  |  |

**Table S4:** Checklist of significant prognostic factors identified by each study

| **Prognostic Factors** | | | Bender 2019 | Berger 1995 | Boozalis 2018 | Freytes 2007 | Herranz 1998 | Huang 2011 | Jing 1999 | Kundu 1995 | Magand 2011 | Maurer 2004 | Motegi 2014 | Unemori 2010 | Queiroz Zancanaro 2005 |
| --- | --- | --- | --- | --- | --- | --- | --- | --- | --- | --- | --- | --- | --- | --- | --- |
| **Patient** | Age | | ✓ | ✓ | ✓ | ✓ | ✓ | ✓ | ✓ | ✓ | ✓ | ✓ | ✓ | ✓ | ✓ |
|  | Sex | | ✓ | ✓ | ✓ | ✓ | ✓ | ✓ | ✓ | ✓ | ✓ | ✓ | ✓ | ✓ | ✓ |
|  | Race | | ✓ | ✓ | ✓ | ✓ | ✓ |  | ✓ | ✓ | ✓ | ✓ | ✓ | ✓ | ✓ |
| **HIV** | CD4 count | |  | ✓ |  | ✓ | ✓ | ✓ | ✓ |  | ✓ |  | ✓ | ✓ | ✓ |
|  | Viral load | |  |  |  | ✓ |  |  |  |  |  |  | ✓ | ✓ | ✓ |
|  | Treatment - HAART | | ✓ |  |  | ✓ |  | ✓ |  |  | ✓ |  | ✓ | ✓ | ✓ |
| **Prurigo Nodularis** | Presentation | |  | ✓ |  |  | ✓ | ✓ | ✓ | ✓ |  |  | ✓ | ✓ |  |
|  |  | Morphology |  | ✓ |  |  | ✓ |  | ✓ | ✓ |  |  | ✓ |  |  |
|  |  | Location |  | ✓ |  |  | ✓ |  |  | ✓ |  |  | ✓ | ✓ |  |
|  | Treatment – Systemic | |  | ✓ |  |  | ✓ |  |  |  |  | ✓ | ✓ | ✓ |  |
|  |  | Thalidomide |  | ✓ |  |  | ✓ |  |  |  |  | ✓ | ✓ |  |  |
|  |  | Corticosteroids |  | ✓ |  |  |  |  |  |  |  |  |  | ✓ |  |
|  |  | Antihistamines |  |  |  |  | ✓ |  |  |  |  | ✓ |  | ✓ |  |
|  | Treatment – Topical | |  | ✓ |  |  | ✓ |  |  | ✓ |  | ✓ | ✓ | ✓ |  |
|  |  | Corticosteroids |  |  |  |  | ✓ |  |  | ✓ |  | ✓ | ✓ | ✓ |  |
|  | Treatment – Phototherapy | |  | ✓ |  |  |  |  |  | ✓ |  |  | ✓ | ✓ |  |
| **Other** | Comorbidities | | ✓ |  | ✓ | ✓ | ✓ |  | ✓ |  |  |  | ✓ |  |  |
|  |  | Neoplastic | ✓ |  |  | ✓ |  |  |  |  |  |  |  |  |  |
|  |  | Infectious | ✓ |  | ✓ | ✓ |  |  | ✓ |  |  |  |  |  |  |
|  |  | Inflammatory | ✓ |  |  |  | ✓ |  |  |  |  |  |  |  |  |
|  |  | Dermatological |  |  | ✓ |  |  |  | ✓ |  |  |  |  |  |  |
|  |  | Psychiatric |  |  | ✓ | ✓ | ✓ |  |  |  |  |  | ✓ |  |  |
|  |  | Cardiovascular |  |  | ✓ | ✓ |  |  |  |  |  |  |  |  |  |
|  |  | Other |  |  | ✓ | ✓ |  |  |  |  |  |  |  |  |  |
